# Can linked emergency department data help assess the out-of-hospital burden of acute lower respiratory infections? A population-based cohort study

**DOI:** 10.1186/1471-2458-12-703

**Published:** 2012-08-28

**Authors:** Hannah C Moore, Nicholas de Klerk, Peter Jacoby, Peter Richmond, Deborah Lehmann

**Affiliations:** 1 Division of Population Sciences, Telethon Institute for Child Health Research, Centre for Child Health Research, University of Western Australia, Perth, Australia; 2 School of Paediatrics and Child Health, University of Western Australia, Perth, Australia; 3 Department of Paediatric and Adolescent Medicine, Princess Margaret Hospital for Children, Perth, Australia

**Keywords:** Emergency department, Outpatient, Acute lower respiratory infection, Data linkage, Children

## Abstract

**Background:**

There is a lack of data on the out-of-hospital burden of acute lower respiratory infections (ALRI) in developed countries. Administrative datasets from emergency departments (ED) may assist in addressing this.

**Methods:**

We undertook a retrospective population-based study of ED presentations for respiratory-related reasons linked to birth data from 245,249 singleton live births in Western Australia. ED presentation rates <9 years of age were calculated for different diagnoses and predictors of ED presentation <5 years were assessed by multiple logistic regression.

**Results:**

ED data from metropolitan WA, representing 178,810 births were available for analysis. From 35,136 presentations, 18,582 (52.9%) had an International Classification of Diseases (ICD) code for ALRI and 434 had a symptom code directly relating to an ALRI ICD code. A further 9600 presentations had a non-specific diagnosis. From the combined 19,016 ALRI presentations, the highest rates were in non-Aboriginal children aged 6–11 months (81.1/1000 child-years) and Aboriginal children aged 1–5 months (314.8/1000). Croup and bronchiolitis accounted for the majority of ALRI ED presentations. Of Aboriginal births, 14.2% presented at least once to ED before age 5 years compared to 6.5% of non-Aboriginal births. Male sex and maternal age <20 years for Aboriginal children and 20–29 years for non-Aboriginal children were the strongest predictors of presentation to ED with ALRI.

**Conclusions:**

ED data can give an insight into the out-of-hospital burden of ALRI. Presentation rates to ED for ALRI were high, but are minimum estimates due to current limitations of the ED datasets. Recommendations for improvement of these data are provided. Despite these limitations, ALRI, in particular bronchiolitis and croup are important causes of presentation to paediatric EDs.

## Background

Bronchiolitis, pneumonia and other acute lower respiratory infections (ALRI) cause substantial morbidity in children. Indigenous populations such as American Indian and Alaskan Native, New Zealand Maoris, Canadian and Australian Aboriginal populations suffer a higher burden than their non-Indigenous counterparts [1-4]. In these studies and many others from developed countries, the estimates of burden are based on hospitalisation rates. There are limitations to using these data to assess the burden of ALRI. Hospitalisations represent the severe end of the ALRI spectrum and therefore underestimate the true burden of ALRI. To prevent transmission and population spread of ALRI, we need to investigate the burden of ALRI at the community level, through general practitioner and emergency services. To our knowledge, there is only one general practitioner dataset, the Bettering the Evaluation and Care of Health, or BEACH, program in Australia [5]. However, BEACH is based on a population sample of general practices in Australia, and not total population-based. While not community-level data, emergency department (ED) data which can be captured through administrative datasets may be able to provide an insight into the out-of-hospital burden of ALRI.

In addition to being a vital component of assessing the burden of ALRI [6], ED data can be used for syndromic surveillance as an early warning of epidemics and to forecast future epidemics [7-9]. For estimates to be meaningful, population-based data (through administrative health datasets) are needed. Unlike some parts of the United States where outpatient data are recorded as part of hospital morbidity datasets [10], ED data in Australia are collected through separate data systems. A single Western Australian (WA) study documented the epidemiological characteristics of ED presentations to four major teaching hospitals in metropolitan WA, but data were not stratified by Aboriginality and focused on upper respiratory infection such as tonsillitis [11]. There are no published data documenting the out-of-hospital burden of ALRI in WA or in Indigenous populations in the developed world.

WA has a strong history of data linkage infrastructure through the Western Australian Data Linkage System (WADLS), one of few such systems worldwide [12]. The WADLS encompasses systematic record linkage and uses probabilistic matching to link records from numerous datasets together for the same individual. The linkage of identifying data is conducted by the data linkage branch at the Western Australian Department of Health and subsequent de-identified data are given to the researcher. ED data are now available as a core dataset within WADLS. Previously we have used linked data from WADLS to investigate the burden, causal pathways and aetiology of ALRI hospitalisations in a WA birth cohort [3,13-15]. Here, our primary aim is to investigate the feasibility of using linked ED data to describe the epidemiology of ALRI presentations to gain insight into the out-of-hospital burden of ALRI. Secondly, we aim to document the factors influencing presentation to ED with ALRI in metropolitan WA Aboriginal and non-Aboriginal children.

## Methods

### Study setting

WA covers one-third of Australia, approximately 2.5 million square kilometres, and in 2009 had a population of 2.2 million [16]. Twenty per cent (approximately 440,000) of this population are children aged less than 14 years, of which Aboriginal children comprise 6%. Three-quarters (74%) of the non-Aboriginal population and 34% of the Aboriginal population of WA live in the metropolitan area which encompasses the capital city, Perth [17].

### Data sources

We conducted a retrospective population-based cohort study of singleton live births born in WA between 1996 and 2005. Data were extracted from the Midwives’ Notification System and Birth and Death Register to form the birth cohort dataset. The Midwives’ Notification System contains information on pregnancy, labour and birth details and is complete for >99% of births in WA. The Birth and Death Register also provides information regarding the date and location of birth and the date and cause of death. Records pertaining to the same individuals from these datasets were linked together through a unique de-identified alpha-numeric code provided by the WADLS. Full details of data extraction and cleaning of the birth cohort data are provided elsewhere [3,13]. In brief, the cohort consisted of 245, 249 singleton live births of which 7.1% were identified as Aboriginal. The Midwives’ Notification System and the Birth and Death Register provided information concerning Aboriginal status and a child was identified as such if at least one record in one of the datasets recorded the child as Aboriginal. This was to minimise any potential underestimation of Aboriginal status. The residential postcode of the mother at the time of her child’s birth was used to classify the cohort to metropolitan, rural and remote births.

### Emergency department data collection

The Emergency Department Data Collection (EDDC) consists of several datasets: the Emergency Department Information System (EDIS – all nine metropolitan hospitals), The Open Patient Administration System (TOPAS – one rural hospital), and the Health Care and Related Information System (most other rural and remote hospitals). Records in EDIS contain one International Classification of Diseases (ICD) version 10 diagnosis code [18] and one symptom code, whereas records from the rural and remote EDs only contain broad level diagnostic categories (e.g. ‘Respiratory’) which cannot be used to identify the specific cause of presentation. Therefore our data extraction was limited to records in the EDIS system representing metropolitan WA. Six of the nine metropolitan hospitals commenced data collection in July 2001, another in July 2002 and the remaining two in 2004/2005. The only dedicated paediatric ED in WA, Princess Margaret Hospital for Children located in metropolitan Perth, commenced data collection in July 2001. This hospital accounts for 55% of ALRI hospitalisations in metropolitan WA (Moore HC, unpublished data). EDIS records were extracted and linked to the birth cohort through the unique alpha-numeric code provided by WADLS if they contained a respiratory ICD diagnosis code or a symptom in the broad classification of ‘respiratory’ from the symptom codes. Presentations with ICD diagnosis codes were further classified into pneumonia (J12-J18), bronchiolitis (J21), croup (J05), whooping cough (A37), influenza (J10-J11), bronchitis (J20) and unspecified ALRI (J22). The symptom codes were further classified into ‘cough’, ‘wheeze’, ‘febrile’, ‘bronchiolitis’, ‘croup’, ‘bronchitis’, ‘chest infection’ or ‘pneumonia’.

### Statistical analysis

Due to the lack of availability of non-metropolitan emergency department data, the birth cohort was restricted to metropolitan births only (172,444 non-Aboriginal children and 6366 Aboriginal children). Using dates of birth and death, a person-time-at-risk denominator was generated for metropolitan-born Aboriginal and non-Aboriginal children for specific age groups in the period 2001–2005. Age-specific ED presentation rates for ALRI were then calculated per 1000 child-years at risk. ED presentation rates were compared between Aboriginal and non-Aboriginal children using incidence rate ratios (IRR) and 95% confidence intervals (CI). The monthly distribution of ED presentations was determined using the month of presentation. The chi-square test was used to investigate differences in proportions of ED presentations across sub-groups.

To examine factors influencing presentation to ED with ALRI, data were restricted to ED presentations in the first 5 years of life. Predictors of ED presentations were investigated using multiple logistic regression for Aboriginal and non-Aboriginal children separately with the binary outcome of at least one ALRI presentation before age 5 years. ALRI presentation was defined as either ICD-coded ALRI or symptom-coded ALRI. In addition to reporting odds ratios (OR) and 95% CIs, we used the *aflogit* command in Stata to calculate population attributable fractions (PAFs) and their 95% CIs. This was to estimate the proportion of the risk of ED presentation with ALRI that can be attributed to the causal effects of the risk factor.

Predictors included those maternal and infant factors recorded on the available datasets: sex, gestational age, number of mother’s previous pregnancies, season of birth, mode of delivery, maternal age, maternal smoking or asthma during pregnancy, percent optimal birthweight (POBW) and the Socio-Economic Index for Area (SEIFA) [19] scores at the time of birth. POBW is a measure which takes into account gestational duration, foetal sex, maternal age, maternal height and parity and is considered more accurate than birthweight alone [20]. SEIFA is comprised of several indices, the main index being the index of relative disadvantage which is derived from low income, low educational attainment, high unemployment and jobs in unskilled occupations. SEIFA scores are measured at the collection district level, the smallest unit available for population-based analyses. Season of birth was derived from the month of birth as follows: summer (Dec-Feb), autumn (Mar-May), winter (Jun-Aug) and spring (Sept-Nov). The models were also adjusted for year of birth. These predictors were chosen as they were found to be significant risk factors of hospital admission with ALRI from our previous analyses [13]. Maternal smoking during pregnancy was recorded from 1997, therefore only children born 1997–2005 were included in the models.

### Ethical approval

This study was approved by the Princess Margaret Hospital for Children Ethics Committee, the Department of Health WA Human Research Ethics Committee and the Western Australian Aboriginal Health and Information Ethics Committee. Use of the data from the WADLS was approved by the WA Data Linkage Branch.

## Results

### Classification of ED presentations

**Figure 1:**
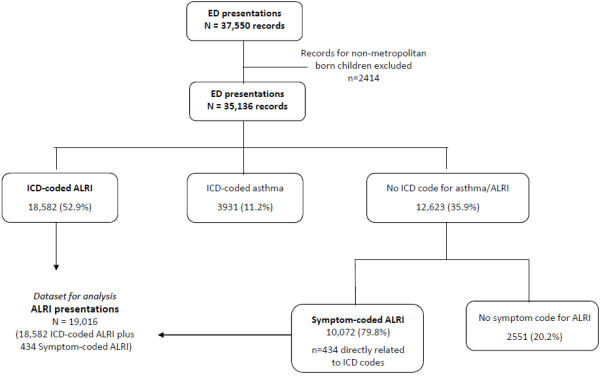
Classification of emergency department presentations.

Over 95% of the extracted ED records for the period 2001–2005 could be linked to the birth cohort dataset. The remaining 4.3% (n = 1670) were presentations for multiple births or births outside WA and therefore not included in our birth cohort dataset. From 37,550 presentations initially extracted, 2414 records were of children born in rural and remote WA or with missing postcode at the time of birth. These records were excluded from the analysis. Figure 1 describes the classification of ED presentations according to diagnosis. Of the remaining 35,136 presentations, there were 18,582 (52.9%) presentations with an ICD diagnosis code for ALRI and 3931 (11.2%) presentations with an ICD diagnosis code for asthma (Figure 1). The remaining 36% of presentations did not contain a specific ALRI or asthma ICD diagnosis code. Of these, there were 10,072 presentations that had a broad symptom code for ALRI (Figure 1) and 2551 presentations for upper respiratory infection or were too broad to be coded (e.g. ICD code for viral infection of unspecified site, or symptom code ‘respiratory’ with no further sub-classification). Of the 10,072 symptom-coded ALRI presentations, 85% had a symptom code for cough, 10% for wheeze and 4.3% (n = 434) had a symptom code directly related to an ICD diagnosis code (‘croup’ relating to ICD code for croup, ‘pneumonia’ relating to pneumonia, ‘bronchiolitis’ relating to bronchiolitis and ‘chest infection’ relating to unspecified ALRI).

Compared to symptom-coded ALRI presentations, ICD-coded ALRI presentations were from younger children (e.g. 37.7% <12 months vs 32.2%; χ^2^ = 88.3; p < 0.001) and more likely to be male (62.2% vs 55.2%; χ^2^ = 132.6; p < 0.001). Additionally, the proportion of presentations in Aboriginal children was lower in those with an ICD-coded ALRI (8.5%) compared to a symptom-coded ALRI (10.2%; χ^2^ = 21.6; p < 0.001).

Due to the non-specific nature of some of the symptom codes, further analyses were limited to those 19,016 presentations that could be classified as pneumonia, bronchiolitis, croup, whooping cough, influenza, bronchitis or unspecified ALRI. These presentations were a combination of ICD-coded ALRI and the 434 presentations with symptom codes that directly related to an ALRI ICD code (Figure 1).

### ALRI presentations

There were 17,399 presentations from non-Aboriginal children of which 62.2% were male and there were 1617 presentations in Aboriginal children of which 60.9% were male. Using population person-time-at-risk denominators, the highest presentation rates for ALRI were in those aged 6–11 months for non-Aboriginal children (81.1/1000 child-years) and 1–5 months for Aboriginal children (314.8/1000 child-years) and the lowest presentation rates for both Aboriginal and non-Aboriginal children were in those aged 5–9 years (Table 1). In non-Aboriginal children, croup accounted for 45.7% of ALRI presentations, bronchiolitis for 29.7% and pneumonia for 17.1%. The remaining 7.5% of ALRI ED presentations were for influenza, whooping cough, bronchitis and unspecified ALRI. However in Aboriginal children, bronchiolitis accounted for a higher proportion of overall presentations (49.7%) than croup (22.1%). The presentation rate for croup was similar in Aboriginal and non-Aboriginal children while presentation rates for bronchiolitis were 4 times higher in Aboriginal children than in non-Aboriginal children and presentation rates for pneumonia ranged from 2 to 13 times higher in Aboriginal than in non-Aboriginal children (Table 1).

**Table 1:** Number and rate of presentations to emergency departments by ALRI diagnosis in Aboriginal and non-Aboriginal children aged <9 years

**Age group**	Number of presentations (rate*)				**Aboriginal vs non-Aboriginal**	
	**Aboriginal**		**Non-Aboriginal**		**IRR**	**(95% CI)**
Bronchiolitis						
<1 month	8	(29.2)	53	(7.3)	4.0	1.6, 8.4
1-5 months	348	(258.4)	2260	(63.0)	4.1	3.7, 4.6
6-11 months	334	(211.1)	2055	(48.2)	4.4	3.9, 4.9
12-23 months	110	(35.1)	707	(8.3)	4.2	3.4, 5.2
2-4 years	<5	(0.4)	85	(0.3)	1.3	0.3, 3.4
5-9 years	0	-	5	(0.02)	-	-
Total	804	(34.8)	5165	(8.1)	4.3	4.0, 4.6
Croup						
<1 month	<5	(3.7)	<5	(0.1)	26.4	0.3, 2070.8
1-5 months	16	(11.9)	233	(6.5)	1.8	1.0, 3.0
6-11 months	53	(33.5)	944	(22.1)	1.5	1.1, 2.0
12-23 months	90	(28.7)	2020	(23.8)	1.2	1.0, 1.5
2-4 years	145	(15.7)	3593	(14.1)	1.1	0.9, 1.3
5-9 years	52	(7.0)	1158	(5.4)	1.3	1.0, 1.7
Total	357	(15.5)	7949	(12.5)	1.2	1.1, 1.4
Pneumonia						
<1 month	<5	(11.0)	6	(0.8)	13.2	2.1, 61.8
1-5 months	30	(22.3)	101	(2.8)	7.9	5.1, 12.0
6-11 months	59	(37.3)	272	(6.4)	5.9	4.3, 7.8
12-23 months	92	(29.3)	926	(10.9)	2.7	2.2, 3.3
2-4 years	102	(11.0)	1216	(4.8)	2.3	1.9, 2.8
5-9 years	30	(4.0)	455	(2.1)	1.9	1.3, 2.7
Total	316	(13.7)	2976	(4.6)	2.9	2.6, 3.3
Total ALRI (N = 19016)†						
<1 month	12	(43.8)	64	(8.9)	5.0	2.4, 9.3
1-5 months	424	(314.8)	2731	(76.1)	4.1	3.7, 4.6
6-11 months	474	(299.5)	3460	(81.1)	3.7	3.4, 4.1
12-23 months	315	(100.4)	3995	(47.0)	2.1	1.9, 2.4
2-4 years	299	(32.3)	5333	(21.0)	1.5	1.4, 1.7
5-9 years	93	(12.4)	1816	(8.5)	1.5	1.2, 1.8
Total	1617	(70.1)	17,399	(27.3)	2.6	2.4, 2.7

*rate per 1000 child-years at risk for Western Australian metropolitan live births 2001–2005.

†Also includes 123 admissions for influenza (<5 Aboriginal, 121 non-Aboriginal); 94 for whooping cough (11 Aboriginal, 83 non-Aboriginal); 247 for bronchitis (19 Aboriginal, 228 non-Aboriginal) and 985 for unspecified ALRI (108 Aboriginal, 877 non-Aboriginal).

**Figure 2:**
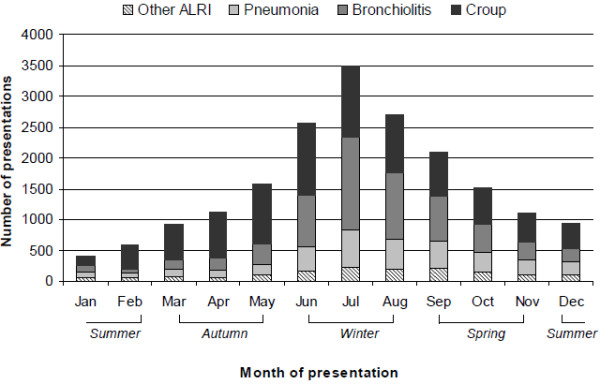
Monthly distribution of presentations to emergency departments for ALRI in children aged <9 years.

ED presentations displayed distinct seasonality with a clear peak in the winter months from June to August (Figure 2). Overall, there were approximately 4.5 times more presentations in winter than in summer. Bronchiolitis displayed the most marked seasonality with 8.9 times more presentations in winter than in summer. The monthly distribution of ED presentations did not differ between Aboriginal and non-Aboriginal children.

Overall, 13,380 children (7.5%) presented to a metropolitan ED for ALRI at least once between 2001 and 2005 before age 9 years. A higher proportion of metropolitan-born Aboriginal children presented at least once (15.2%) compared to metropolitan-born non-Aboriginal children (7.2%; χ^2^ = 566.4; p < 0.001). Of those non-Aboriginal children who presented at least once, 75% presented only once, 17% presented twice, 5% presented 3 times and 3% presented 4 or more times. Of those Aboriginal children who presented at least once, 63% presented only once, 23% twice, 7% 3 times and 7% presented 4 or more times.

### Predictors of ED presentation

To investigate maternal and infant factors influencing presentation to ED, we restricted the dataset to ALRI presentations before age 5 years. Of Aboriginal metropolitan births, 14.2% presented at least once to ED before age 5 years compared to 6.5% of non-Aboriginal births (χ^2^ = 583.54; p < 0.001). For both Aboriginal and non-Aboriginal children, the factor with the largest PAF for ED presentation was male sex (Table 2). Other significant predictors for ED presentation for non-Aboriginal children were being born to a mother with more than one previous pregnancy, born to a mother with asthma during pregnancy, born in autumn, delivered by elective caesarean, born less than 36 weeks gestation and maternal age between 20 and 29 years. For each of these factors, PAFs ranged from 3-8% (Table 2). The only significant predictor in addition to male sex for ED presentation in Aboriginal children was maternal age, where being born to a mother <20 years accounted for 8.4% of the PAF (Table 2).

**Table 2:** Maternal and infant predictors of presentation to metropolitan emergency departments with ALRI in children aged <5 years

**Risk factor**	**Aboriginal**		**Non-Aboriginal**	
	**OR (95% CI)**	**PAF (95% CI)**	**OR (95% CI)**	**PAF (95% CI)**
Male	1.57 (1.32, 1.86)	18.8 (11.5, 25.4)	1.53 (1.46, 1.60)	19.5 (17.5, 21.5)
Gestational age <36 weeks	1.26 (0.99, 1.62)	2.5 (−0.3, 5.2)	1.62 (1.50, 1.75)	3.4 (2.8, 4.0)
Maternal smoking*	0.98 (0.83, 1.17)	-	1.15 (1.08, 1.21)	2.4 (1.4, 3.4)
Maternal asthma*	0.96 (0.75, 1.23)	-	1.38 (1.29, 1.47)	3.6 (2.8, 4.4)
Percent optimal birthweight				
Low <85%	0.95 (0.77, 1.18)	-	1.00 (0.93, 1.07)	-
Normal 85-114%	Reference		Reference	
High ≥115%	1.10 (0.81, 1.50)	0.6 (−1.5, 2.7)	1.07 (1.00, 1.15)	0.7 (−0.01, 1.4)
Previous pregnancies				
0	Reference		Reference	
1	0.97 (0.75, 1.27)	-	1.13 (1.07, 1.20)	3.3 (1.8, 4.9)
2	1.00 (0.74, 1.38)	0.1 (−3.7, 3.8)	1.30 (1.22, 1.39)	4.3 (3.2, 5.4)
≥3	1.29 (0.97, 1.71)	7.7 (−1.0, 15.7)	1.38 (1.28, 1.47)	5.4 (4.3, 6.6)
Season of birth				
Spring	Reference		Reference	
Summer	1.14 (0.90, 1.45)	2.6 (−2.2, 7.2)	1.13 (1.06, 1.20)	2.7 (1.3, 4.0)
Autumn	1.10 (0.87, 1.40)	2.0 (−2.9, 6.6)	1.23 (1.16, 1.31)	4.7 (3.4, 6.1)
Winter	1.08 (0.85, 1.38)	1.5 (−3.1, 6.0)	1.10 (1.03, 1.17)	2.0 (0.7, 3.3)
Mode of delivery				
Vaginal	Reference		Reference	
Instrumental	1.01 (0.71, 1.43)	0.05 (−1.9, 2.0)	1.01 (0.94, 1.08)	0.08 (−0.7, 0.9)
Elective caesarean	1.15 (0.85, 1.55)	1.0 (−1.3, 3.2)	1.25 (1.18, 1.32)	3.4 (2.4, 4.3)
Emergency caesarean	1.18 (0.92, 1.50)	1.9 (−1.1, 4.8)	1.12 (1.05, 1.20)	1.4 (0.6, 2.2)
Maternal age				
≥35 years	Reference		Reference	
30-34 years	1.09 (0.73, 1.63)	0.9 (−3.4, 5.1)	1.10 (1.03, 1.18)	2.6 (0.9, 4.4)
25-29 years	1.45 (1.00, 2.10)	7.1 (0.2, 13.6)	1.28 (1.19, 1.37)	6.1 (4.4, 7.7)
20-24 years	1.39 (0.95, 2.03)	7.0 (−0.9, 14.4)	1.47 (1.36, 1.59)	5.2 (4.1, 6.3)
<20 years	1.69 (1.10, 2.57)	8.4 (2.1, 14.3)	1.82 (1.62, 2.04)	2.4 (1.9, 2.9)
Socio-economic index†				
91-100%	Reference		Reference	
76-90%	0.66 (0.22, 1.98)	-	1.01 (0.92, 1.11)	0.2 (−1.1, 1.4)
26-75%	0.92 (0.35, 2.43)	-	1.09 (1.01, 1.19)	3.8 (0.5, 7.0)
11-25%	1.27 (0.48, 3.34)	6.3 (−21.1, 27.5)	1.30 (1.19, 1.42)	4.2 (2.8, 5.5)
0-10%	1.01 (0.38, 2.68)	0.3 (−25.8, 21.0)	1.23 (1.11, 1.37)	1.7 (0.8, 2.5)

*During pregnancy. Relative to no maternal smoking/asthma.

†91-100% is least disadvantaged and 0-10% is most disadvantaged.

Based on 4105 Aboriginal and 117,989 non-Aboriginal births.

Adjusted for year of birth. Population attributable fraction was not calculated where OR < 1.

## Discussion

We have attempted to provide an insight into the out-of-hospital burden of ALRI in a cohort of Aboriginal and non-Aboriginal children using linked ED data. Bronchiolitis and croup were the most common diagnoses given to children, there was a clear seasonal peak of presentations in winter, and those aged <12 months had significantly higher presentation rates than older children.

We noted more ED presentations for croup than for bronchiolitis in non-Aboriginal children, despite bronchiolitis being the main reason for hospitalisation with ALRI in this population [13]. This is most likely due to the use of steroids over the last two decades in children presenting with croup in WA, who are then likely to be treated as outpatients with very few being admitted [21]. The presentation rates for croup were similar in Aboriginal and non-Aboriginal children. Overall, metropolitan-born Aboriginal children presented to ED with ALRI more often and more frequently than non-Aboriginal children, highlighting the continuing disproportionate burden of ALRI that Aboriginal children in WA suffer: 1 in 6 metropolitan-born Aboriginal children attended an ED with ALRI at least once before their ninth birthday as opposed to 1 in 14 metropolitan-born non-Aboriginal children.

We concentrated on infant and maternal predictors of ED presentation that were identified as risk factors for ALRI hospitalisation from our previous analyses [13]. We restricted this analysis to metropolitan-born children as those children being transferred from rural or remote areas to a metropolitan ED may have a more severe ALRI or have different family circumstances than children attending ED who live in Perth. Consistent with our previous analysis, we also reported predictors of ED presentation separately in Aboriginal and non-Aboriginal children due to the differences in disease burden and risk factors to hospitalisation with ALRI [13]. For all children, male sex and maternal age <30 years were the strongest predictors of ED presentation for ALRI. For non-Aboriginal children, there were many other important factors influencing presentation, such as previous pregnancies, autumn-births and elective caesarean delivery which we identified and offered explanations for in our previous work using hospitalisation as the outcome [13,22]. For Aboriginal children, there were no significant predictors to ED presentation aside from male sex and being born to a teenage mother. This is in contrast to additional risk factors for hospitalisation for ALRI which included low optimal birthweight and maternal smoking during pregnancy [13]. These results suggest that the factors influencing admission to hospital with ALRI are different to the factors influencing presentation to ED in Aboriginal children. Also, importantly, in contrast to our previous analysis of hospitalisation with ALRI, socio-economic status was not a significant predictor of ED presentation in either Aboriginal or non-Aboriginal children, which may be due to the analysis being restricted to metropolitan births. A data linkage study of ED visits in infants from one jurisdiction in the United States found insurance status at birth to be the biggest predictor of ED visits for any diagnosis [10].

The ED presentation rates we have reported here provide us with an estimation of the burden to EDs with ALRI and an insight into the out-of-hospital burden of ALRI. However, our rates presented here are still likely to underestimate the true burden of ALRI to EDs, and are therefore minimum estimates, due to several important limitations of the available ED datasets. Indeed our ED presentation rates for ALRI were lower in all age groups than those reported in a similar study in Boston [6]. First, the EDDC from the nine metropolitan EDs contains only one ICD diagnosis code. Second, data being collected in rural and remote departments cannot be used to identify or differentiate between specific ALRI diagnoses because of the very broad and limited diagnostic categories available. Furthermore, of those metropolitan records with the capacity to record an ICD diagnosis code, this was often missing or too broad to be clinically meaningful for analysis. For example, we identified only 131 ED presentations for influenza from 2001 to 2005 which is an underestimate according to our previously analysed virology data from Princess Margaret Hospital for Children that identified 1802 specimens tested and 199 positive for influenza virus from 2001 to 2005 in children presenting to ED (Moore HC, unpublished data). A previous study in WA using the EDDC has suggested that there is no systematic bias in the failure to record a discharge diagnosis [23]. However, here we have shown differences between those presentations with symptom-coded ALRI (that often had missing ICD codes) and ICD-coded ALRI with respect to Aboriginality, sex and age, suggesting some level of systematic bias of missing ICD diagnosis codes.

Third, based only on one diagnosis code, or the primary discharge diagnosis code, there is a greater chance of inconsistent recording of diagnoses between various EDs. This was the experience in a data linkage study in the United States where the classification of the ALRI diagnosis was partly dependent on which ED children attended in the same geographical area [24]. Other ED registers worldwide have the capacity to record 3 to 10 ICD diagnosis codes [25,26]. The allowance for multiple diagnosis codes increases the amount of diagnostic information in order to identify specific causes of presentation. While we acknowledge that we restricted our dataset by excluding broader or non-specific codes (e.g. viral infection of an unspecified site, which is most likely to be a respiratory infection) we opted to maintain a high specificity in identifying ALRI presentations.

The fourth limitation of these data relates to the representativeness on a population level. Our dataset included nine EDs from metropolitan Perth. While our results presented here accurately reflect the burden of ALRI ED presentations in metropolitan-born children in WA, we cannot extrapolate these findings to rural and remote WA. Additionally, due to the staggered entry of the nine metropolitan EDs to the EDDC system, we have been unable to investigate presentation rates over time, and ED presentations to the hospitals that did not commence data collection until 2004/2005 will have been missed. However, as the data in more recent years are complete, temporal trends will be possible for future data extractions and analyses.

Although, to our knowledge, data from the EDDC system in WA have not been validated against medical records, there are examples of administrative data being used to gain a picture of out-of-hospital burden of ALRI with few concerns over data quality. In one study, there was an overall lack of agreement between discharge diagnoses from administrative ED data and medical chart review but agreement was high for croup (90.4%), pneumonia (86.5%) and bronchiolitis (84.9%) [25].

## Conclusions

We acknowledge that data reporting to EDDC needs to be improved as in its current form it has restricted ability to provide accurate estimates of ED attendance and an insight into the out-of-hospital burden at a population level. We offer the following recommendations: first, similar to coding of hospital admissions throughout the state [27], trained clinical coders should enter ICD diagnosis codes in an effort to reduce the amount of missing data. Second, the capacity to record multiple ICD diagnosis codes should be introduced. Third, rural and remote ED datasets that currently record data on broad level diagnostic categories should migrate to an ICD diagnosis system, as occurs with the hospital morbidity data from rural and remote areas. This would standardise diagnostic coding throughout the state and result in ED data coding being in-line with hospital morbidity coding. These recommendations for improvements to the EDDC have also been made by researchers investigating child maltreatment [28]. Other jurisdictions wanting to develop or improve existing ED data sources should ensure that these recommendations are met. Due to the population-based data linkage infrastructure in WA and the increasing ability to link other datasets such as microbiology records [15], these improvements are justifiable. If achieved, ED could be a useful data source in WA to provide measures of the out-of-hospital burden, in light of the lack of population-based general practitioner data, and improve surveillance systems to detect outbreaks of future infections.

## Competing interests

The authors declare that they have no competing interests.

## Authors’ contributions

HCM conceived the idea for this analysis, performed all of the data manipulation and data analysis and wrote the first and subsequent drafts of the manuscript. NdK and PJ assisted with statistical analyses and interpretation of the data. DL and PR assisted in the study design, the interpretation of the results and critically revised drafts of the paper. All authors read and approved the final version of the manuscript.

## Pre-publication history

The pre-publication history for this paper can be accessed here:

http://www.biomedcentral.com/1471-2458/12/703/prepub
